# Intelligent Health: Progress and Benefit of Artificial Intelligence in Sensing-Based Monitoring and Disease Diagnosis

**DOI:** 10.3390/s23229053

**Published:** 2023-11-08

**Authors:** Gabriela Palavicini

**Affiliations:** Department of Media and Digital Culture, Instituto Tecnológico y de Estudios Superiores de Monterrey, Mexico City 01389, Mexico; gpalavicini@tec.mx

**Keywords:** biosensors, technology, medicine, artificial intelligence

## Abstract

Technology has progressed and allows people to go further in multiple fields related to social issues. Medicine cannot be the exception, especially nowadays, when the COVID-19 pandemic has accelerated the use of technology to continue living meaningfully, but mainly in giving consideration to people who remain confined at home with health issues. Our research question is: how can artificial intelligence (AI) translated into technological devices be used to identify health issues, improve people’s health, or prevent severe patient damage? Our work hypothesis is that technology has improved so much during the last decades that Medicine cannot remain apart from this progress. It must integrate technology into treatments so proper communication between intelligent devices and human bodies could better prevent health issues and even correct those already manifested. Consequently, we will answer: what has been the progress of Medicine using intelligent sensor-based devices? Which of those devices are the most used in medical practices? Which is the most benefited population, and what do physicians currently use this technology for? Could sensor-based monitoring and disease diagnosis represent a difference in how the medical *praxis* takes place nowadays, favouring prevention as opposed to healing?

## 1. Introduction

This paper explains the importance of using new technologies, mainly artificial intelligence translated into biosensors, to monitor and diagnose illnesses, avoid severe consequences of different disorders in patients, and help physicians provide better attention with quicker diagnosis and treatments. Biosensors are not new; the first one dates back to 1950 with Leland C. Clark’s development of oxygen detection [1], currently known as the Father of Biosensors [2], and introduced this concept into Medicine. He was aided by new technology to help them evolve during all these decades, and also impulsed by the advancements in nanomaterials and fabrication strategies [3].

When we refer to biosensors, we need to know that different kinds exist according to their complexity; from those only used for informing people are oximeters, those providing a mediator between the sensor and the answer are enzymatic ones, such as glucometers, or even those providing drugs or intervening as a pacemaker to avoid a patient’s death [4]. Despite this, they constitute a large group of new tools to potentialise human capacities: the physicians and the patients in self-care and the communication between both. Biosensors have unlimited potential: “as it is easy, scalable and effective in manufacturing processes” [5]. Nevertheless, their categorisation according to their powers will facilitate their use.

What must be remembered is that a biosensor is mainly an analytic tool [6]. In this sense, its use for diagnosis and prognosis becomes significant when considerable data amounts are always present and in any sphere. The COVID-19 pandemic has shown the importance of processing those amounts of data in the shortest periods, and biosensors’ role in the epidemic was significant [7]. Considering this, they can become an effective tool for improving Medicine in monitoring, diagnosis, and prescription, mainly in critical areas, illnesses, and stages [8]. At the same time, biosensors can, nowadays, contribute, helped by new technologies such as artificial intelligence, to improving a patient’s quality of life due to aiding in prevention and healthcare [9]. 

However, since biosensors are mainly data analysers, data culture is also needed in both populations: those suffering from a medical disorder and those providing health services to adopt new ways of proceeding. Considering that Medicine is the most human profession and it “must contribute to benefit society” [10], it needs to anticipate and foresee the future damages a patient could face with his medical history. Biosensors can intervene and provide drugs, but they can also help in monitoring and prevention, rather than healing [11]. It is essential these days to consider monitoring as one of the main activities to pursue in medical practice to prevent and avoid severe health issues in entire populations and create a self-care habit, even if we are not yet there and if technological advances need to continue being developed.

In the next section, we present the method in a very brief way. In section three, the literature review introduces the technological progress from a general point of view, the role of data and their importance in the medical field from a social perspective, and biosensors’ history as a basis to lay the foundations further to understand their utility and significance in medical applications. It shows their interrelationships and the need for a culture change. Deleuze’s deontology considers biology and virtuality, too [12]. His “socialisation of the machine” [13] is also evoked to understand and reinforce the need currently and constantly in demanding different devices to improve the patient’s life.

In section four, the paper presents the use of technology in medical practice, alluding to the importance of artificial intelligence and an overview of the progress in Medicine, such as some practices using biosensors in imaging, metabolic monitoring, neurological treatments, and cardiovascular implementations, without being an exhaustive revision.

In the fifth section, a discussion of findings after the overview takes place, and in the sixth section, some conclusions are presented.

## 2. Method

This paper is a descriptive study of what has been done regarding biosensors for monitoring and diagnosing to understand the importance of their use. It analyses its advantages and the use of technology, specifically AI, in this kind of tool to be more efficient in clinical attention and treatments. The paper considers a qualitative approach based on a literature review and the deductive method. In this sense, we reviewed the technological progress and the importance of a data culture to present further the advances in their use in some medical areas. 

## 3. Literature Review

### 3.1. Technological Progress and the Role of Data in the Medical Field

In recent times, one of the most developed areas has been technology. The COVID-19 pandemic has accelerated and expanded its use and presence in some areas where it could be delayed [14]. We find many activities, from socialising, learning, teaching, and even healing. We could not imagine technology as the main element in achieving them three years ago, even if it has been present from the beginning of humanity until now. But reality has shown that with it, all activities would be possible to undertake. Other associated elements of technology that are not necessarily visible are also emerging. Consequently, technology in a communicational era has become indispensable [15], despite its immediacy and lack of permanence. However, it is not looking for new inventions that could transform reality in terms of the presence of a “Singularity”, according to Kurzweil [16].

In the case of biosensors, communication intervenes when another path alters the continuity of the system (a body) as conceived but allows continuity in another human’s development phase. They are the link between the patient’s body and the physician. As a bridge, they make possible a better diagnosis and treatment equivalent to chaotic events which refer to a rupture. In this case, another path alters the continuity of the system as it was conceived but allows continuity in another human’s development phase. This means that: “they (catastrophic events) no longer extract a structure common to any elements whatever, they spread out an event -to move about- they counter effectuate an event which cuts different bodies and is effected in various structures” [17] to increase the body’s capacity of regeneration, intervening when necessary or even healing. In these times, virtuality and immaterial are more present than ever, but they must also be tangible. How can technology be part of tangibles, invisible, and contained in a supernatural world, allowing evolutionary but current processes to attain high-quality development levels? Under these circumstances, we can affirm its presence and progress parallel to people’s evolution, such as countries’ development.

The perspective has changed due to technological advances, and the needs they present, considering their role in the future, are undeniable. Technology did not have to wait for its steady evolution. Following what McShea stated, “biological evolution also applies to technology: insufficient credible evidence exists to make a forceful empirical case for or against the complexity thesis [18]. In this case, a health crisis allows an abrupt positioning in societies. This shows that its’ improvement is closely related to economic, social, and political changes or needs and that they are not dissociable nowadays”. And, even if humanity had experienced this over time, it still seemed to be separated [19].

Analysing technological and social advances must be done from a holistic perspective, which means considering a systemic point of view [20]. All parts are interrelated and affect the others with the corresponding impact. “The difficult part is making all the elements of a non-homogenous set converge, making them function together” [17]. This idea refers to another Deleuze reference about diversity, “which focuses on unification, centres of totalisation. […] It is a being-multiple, instead of a being-one, a being-whole or being as subject”.

In consequence, all the parts intervene in the whole system’s development. Volti also considered this when he affirmed: “The technological change has been an important force in social and institutional configuration roles, even if its development has been the result of human actions, taking place in a particular social environment” [21]. There must be a synergy between all the elements involved: people and their needs, the economic contexts allowing technical and technological advances according to those needs or even getting ahead, and the political contexts enabling citizenship and focusing on existing areas, e.g., demanding investment in scientific development according to people’s demands. 

None of these aspects could act alone, even if we used to think of technology as being small steps and creating gadgets to make life easier or generating new inventions to replace everyday products, as it happened at the time with media. The cell phone is the best example of returning all the revolutionary effects of their time, such as television, telephone, and cameras. We cannot deny what Scherer affirmed in this sense at the beginning of this century: “Technological change -would be able- to modify, in the future, emotions and people’s behaviour” [22]. 

That is happening twenty years after his affirmation. Media are nowadays the best example of Deleuze’s multiplicities [17]. And, at this time, media are the “between” that allows the needed communication among those relations and between a tangible world and an intangible one. But, once again, media was developed following social needs. 

Therefore, we will even say that communication constitutes Deleuze’s “between” compared with Pollock’s lines [17] because of the possibilities of bifurcations and the need for its presence. Communication is always present in each system; it can also be understood as the connection and interrelation between and among different elements in different processes and stages. This is the case with biosensors, which establish communication between the bodies and the device by codes [23]. Those codes allow transmission, and at the same time, they constitute a form of communication [24]. Those connections are infinite because of the existing spheres with multiple bifurcations. Those bifurcations have attained fields that people never thought could be immersed in such technological change as it is in Medicine. 

Nevertheless, it is impossible to remain apart without being left behind; Medicine and its study object, the body, stay in the middle, evoked by Deleuze since Spinoza’s first principle “is a single substance for all the attributes” [17] between progressing and using new advancing technologies or more conservative treatments and praxis. This is why, before going further, we need to make a quick reference to biosensors’ history since this has been an area that, as much as technology has progressed, has also developed since they saw the light and they have not been left behind. On the contrary, they have also improved during the last years to allow better learning, more specific monitoring, better data storage, and even intervention in people’s health when necessary to avoid death. 

#### 3.1.1. Biosensors’ History

Biosensors’ historical evolution can be explained in four generations or stages, each contributing further knowledge to the biosensors known today. The beginning of the concept referred to the combination of enzymes and electrochemical sensors, mixing biological elements with electrochemical research [25].

As mentioned, the father of biosensors is currently known to be the biochemist Leland C. Clark Jr.; his discovery began as a failed sensor that tried to measure oxygen reduction with an electrode’s surface. Thus, the goal was to determine blood oxygenation and revolutionise the medical field as it would answer glucose problems. Nevertheless, it was unsuccessful due to the blood components being absorbed, which distorted the signal [26] and as a consequence, he did not reach his objective at the first trial. 

Afterwards, the biochemist had: “the ingenious idea of using the cellophane wrapper of a cigarette packet on his sensor” [27], eventually having success with his invention, which was then called the “Clark electrode” [28]. Alongside Clark’s triumphant experiment, another innovation in the history of sensors was the potentiometric urea electrode [29]. Both constituted first-generation biosensors.

In the second generation, necessities shifted to simplify the process and to improve the analytical quality. To do that: “auxiliary enzymes and co-reactants are co-immobilised with the analyte converting enzyme” [30]. Overall, the objective was to solve first-generation issues, in particular by incorporating a synthetic mediator (known as an electron shuttle molecule) that could replace the dissolved O2 and finally facilitate electron transfers, automating how biosensors work [31]. 

Regarding third-generation biosensors, they embodied the union of a wiring of an enzyme to the electrode in a conductive way. It was a relevant advancement as: “immobilised mediators allow efficient electron transfer, resulting in a higher current density” [32]. It also included the involvement of biomolecules with biosensing material, such as using Surface Plasmon Resonance (SPR) [33].

**Figure 1:**
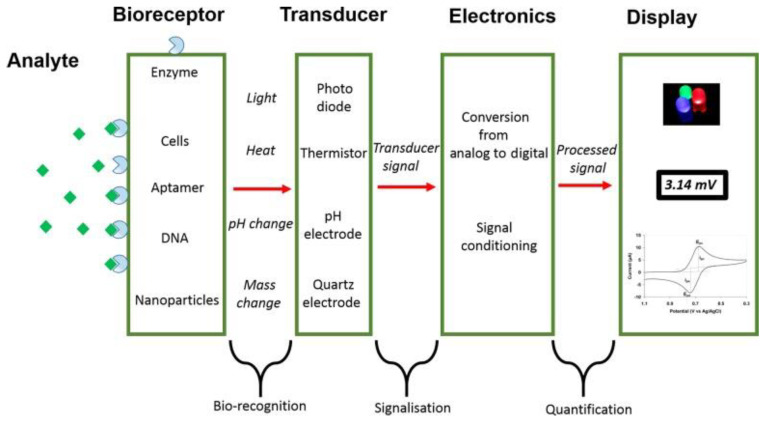
Schematic representation of a biosensor.

Finally, during the fourth-generation of biosensors, electron transfer with potent electrocatalytic activity was employed. Nonetheless, it still had several complications: “for commercial use in monitoring patients with diabetes, such as poor selectivity and the requirement for alkaline condition during analysis” [34]. In this critical situation, the importance of studying biosensors is still current and mandatory in Medicine and is also encouraged by technological advances. Altogether, the disciplines considered throughout this paper could merge: “the diagnostic capabilities of biomedicine with modern technological advances in microelectronics, optoelectronics, and nanotechnology” [35]. In Figure 1 [1], we can appreciate the schematic representation of a biosensor:

#### 3.1.2. The Importance of Working with Data Sets for the Betterment of the Medical Practice

Technology has been improving slowly in some specific areas, even during the last few years, in a similar way as human nature, and the evolution mentioned before; considering that human factors are slow to change, so is technology [36]. It has been developed according to science, which is another field where improvement needs time because of maturation processes, tests, and data set collection. In this sense, those theories consider “science in its development thanks to successive episodes of rupture which introduce new paradigmatic ways to conceive the world, or in other terms thanks to successive revolutions able to provoke” [19] “changes with an impact in the subsequent scientific practice” [37], as it was observed in vaccine progress resulting from the pandemic. “Science is -nowadays- becoming increasingly event-centred [événementielle] instead of “structural”, which leads to a different way of living science from scientists who are more and more concerned with singular events, of an incorporeal nature, which is affected in bodies, in states of bodies, in completely heterogenous assemblages” [17]. Nevertheless, people did not tend to think of technological advances as something that could be essential in their lives despite artificial intelligence and robotics expansion [38]. From this perspective, AI has become critical for promoting and generating new ways of working and conceiving the world during the last few years [39].

The world has focused on some elements that are not necessarily evident. Nevertheless, they constitute the core of every current operation and daily functioning in all areas where human beings interfere. Like all our daily interactions with other human beings, things, or processes, technology implies invisible but present intangibles; without them, things could not be possible. That way, technology may become a part of what Liu calls “intangible cultural heritage” [40], becoming part of human wisdom and experiences necessary to continue societies. Technology determines culture and vice versa in that technology represents the means to express and create a culture, and this last factor evolves thanks to technological artefacts. At the same time, culture and technology emerge from human needs to cover humanity’s necessities and support its development to obtain a better quality of life, as “Societies draw on innovation to solve problems” [41].

We are talking about all those data amounts needed to reproduce a sequence. Or, in a more current daily operation or activity, predict consumer behaviours and body reactions in a more sensitive area. And, even if it could seem very reductionist to the end of this paper and the theme we are treating here, human beings themselves are data. Human beings’ bodies, chemical reactions, biological functioning, and concrete actions generate data sets. All their daily activities leave a trace that can be read in a data set to understand and prevent their subsequent behaviours, decision-making processes, and even their possible health history, which could be modified in advance. This is why AI has become critical for promoting and generating new ways of working and conceiving the world during the last few years, as we will see further.

Once, Spinoza questioned, “What can a body do?” [17]; nowadays, humanity is in the presence of taking that power he evoked, helped by artificial elements. And, in this sense, we could change his perspective. Instead of considering the affections empowering or diminishing the body’s capacity in terms of feelings of joy or sadness [17], let us assume the biological dimension, referring to the components of a body, and retake his idea of “its [the body] capacity as a result of the relationships of which it is composed” [17] with its internal and external world; there are affectations in both and a collective impact. The equivalent to Spinoza’s elements is illness for sadness and health for joy, with the same effects.

Nevertheless, scientists have been, for many years, collecting information about people’s body functioning. Still, there is not yet a solution to approach that amount of information and use it to benefit the person, mainly where health imbalances occur [42]. This is why working on those data sets is essential to make intangibles tangible using analytical devices. Scientists must consider how data sets can be used as inputs to improve Medicine and health care. They represent the raw material and can provide information according to the physician’s use in decision-making processes [43]. Data sets can be used to evidence a patient’s clinical history and variable reactions, such as drugs, complete treatments, affectations to specific organs, and a biochemical organ reaction following other affectations [44]. In this sense, their utility must be focused to know their use for helping a patient to heal or prevent illness, which would be the desired action. 

Data sets can be considered in three dimensions [45]: (a) improvement in attention following previous findings, which would allow faster engagement based on previous knowledge, which leads to speedier clinical attention; (b) the prevention of other illnesses the patient could be predisposed to based on prospective clinical history, and (c) healing based on records of how the patient manifests during long periods of follow-up. Even if there is needed a cultural change regarding health, from the point of view of both populations: patients, who mainly visit the doctor when they feel ill, and doctors focused on healing, from this new perspective, in a basic query, counting on easy access to databases will allow physicians to be well-informed and to act immediately, avoiding the increasing harm a patient can manifest due to chemical imbalances or injuries. Under this approach, institutions must consider counting on updated data or, for example, obtaining data from electronic health records [46]. Updated evidence databases will provide immediate help to physicians and patients.

Time is essential when referring to health and its detrimental consequences in people’s lives. An example of actions that can be carried on, associated with the first dimension, which follows previous findings, is the Support System to Clinical Decisions (SADC) database developed by Wolters Kluwer, which accompanies the patient’s clinical digital history. “A published report from the Institute of Medicine (IOM) identified the contribution of a SADC based on evidence as one of the eight main functions of offering digital clinical histories to ensure a higher level of security, quality and effectiveness when offering medical attention” [47].

At the same time, those systems contain logarithms and mathematical functions that, combined with the patient’s clinical history, provide much more precise information about steps to follow, which could contribute to preventing an illness from emerging or healing the patient when the sickness has manifested. Databases can be as complete and complex as one wants. Still, they can also benefit from general information like the one we mentioned, which contains imaging and laboratory results which complete the patient’s medical history [48]. 

During the last few years, there has been an explosion in the use of big data in public health. The increasing of support in the European Union is an example, as it is shown in Table 1 [49]:

The increase in use and digitalised information allow access to multiple databases for diagnosis, with a growing tendency [50] helped by AI devices analysing it. Data nourishes biosensors and they become tangible. This propensity will allow faster results in less time. Still, it may need effort to change the classical practice and the medical culture, adopting them in daily activity. It could seem that more work feeding the databases will be given later. DesRoches et al. mentioned that “there must be a meaningful use as: including the adoption of certified electronic health record systems, the ability to engage in electronic prescribing, the ability to exchange information, and the ability to report on clinical and other quality measures” [51]. This means that information must be available to any user and action, the patients, and the physicians, at any time to develop competencies in each area. 

There also must be pre-conceived and implemented strategies oriented to using those data in the sense that institutions need to construct the infrastructure required to work with them, which is the same as talking about the “democratisation of data” [52]. But, it is essential to remember that even though we have said that use is still far from a daily way of acting, it does not represent more than the basis to settle a change in the use of technology in the clinical field and the general medical area. Those actions are only the beginning of what could be the birth of a new era and a massive improvement in Medicine, knowing what to do with all the information collected and how to use it according to institutionally implemented strategies and objectives.

People, physicians, and patients need to be convinced that, nowadays, technology can help with the amounts of new information generated worldwide related to many medical fields, as well as with the benefits it can provide [51]. “The accumulation of data makes open-minded observers converge on the truth and come to agreement” [51]. These agreements may be the more prosperous result related to the prognosis of illness behaviour in a general perspective. 

Those large data sets provided by patients during long periods would allow a quick response to diagnosis and prevent epidemics, which is understood as a spreading illness worldwide, even if it is not contagious in strictum sensu as is diabetes or obesity, a fortiori in pandemic infectious cases [53]. They would also allow an understanding of illnesses’ behaviour to find cures, such as metabolic or neurological diseases. In this sense, if we conceive groups of conditions considering the area they belong to and group them in databases, information could be found more accessible for better attention. The best example we can see now is the SARS-CoV-2 vaccines which needed a high consensus about their testing, development, and efficacity to prevent mortality by this virus, allowing a quick response in their manufacturing due to sharing the knowledge that was acquired [54].

Information is nothing else but data. Data culture is needed in clinics, physicians’ daily practice, and patients [55]. Since a data-driven culture permeates people and mainly this area, the decision making would change and, based on everyday practice, will simplify physicians’ tasks such as diagnosis and medication with higher benefits in the sense of reaching more elevated amounts of people, but also, the main point, the quality of the provided service with fewer percentages of mistakes and higher amounts of people healed. 

Benefits could be for everybody and even the entire world, allowing us to prevent, as far as possible, a pandemic like the one humanity has faced since March 2020. “After 70 studies, it was found that clinical decisions-system support improved the clinical practice in 68% of patients, and (…) indicators in the medical practice show that the improvement (…) included decisions based on a computer system” [47]. Still, it implies changes in how medical procedures occur nowadays without losing the human side and without conceiving a patient as only a data source.

This problem can emerge and must be prevented. Patients are data and thinking of them in this sense helps clinical attention. Still, since they are mainly human beings, this element cannot be forgotten when looking for care improvement which is supported by innovations [56]. This means that even if technology is there and its use becomes indispensable to accelerate decision-making processes, the quality of care would still depend on physicians’ care for holistic improvement in the medical area. To facilitate physicians and medical institutions’ work, they must derive a higher quality of treatments and care; technology and databases per se have no impact on patient health and the health system’s improvement, as DesRoches et al. established [51]. At the same time, technology and data must be focused on specific treatments to get an absolute advantage of technology. As [57] established: “The separate realms of “wellness tracking” and “disease self-monitoring” and “activity data” and “medical data” are thus blurred, which is somewhat mirrored in an increasing prominence of concepts such as “patient-generated health data” and “personal health technology” where the focus is on the individual producer of data, rather than on the specific context or purpose of use”.

Thus, the proper use of AI would enable physicians to provide the right treatment to the right patient at the right time. It will help with categorising data and analysing them in separate groups of patients according to symptoms, illnesses, previous treatments, and their negative and positive responses. Clusters can be formed considering other variables such as age, clinical history, and race to allow a more effective use of time using data management. This could seem simple to do; however, to attain these levels, there needs to be a focus on a data-driven culture but also on what the medical staff want to achieve by developing some processes that, step by step, lead to counting on AI in most methods, which is not replacing people but instead potentializing human capacities and helping them in a more focused way, as we will see in the next section.

## 4. Technology in the Medical Practice

### 4.1. The Innovative Medicine of the Future: The Importance of Artificial Intelligence and an Overview of the Progress in Medicine

In this section, we will describe the importance of AI in Medicine and the use of some AI applications that are already present, including their impact on attention and prevention using monitoring and diagnosis biosensors. As [58] said: “AI can provide substantial improvements in all areas of healthcare from diagnostics to treatment”. These actions are the beginning of a “revolutionary becoming” in Medicine. The information provided by patients over long periods of time would allow a quick response to diagnostics and prevent epidemics. Groups of conditions could be formed considering their area and information could be seen as more accessible for better attention. This is why AI devices translated into biosensors will help physicians monitor and diagnose based on those data.

AI devices could improve more and more diagnostics, prevention, intervention, and healing other than X-ray and general imaging where it has enhanced diagnosis in shorter periods for the benefit of the patients. AI has also eased treatments and their planning without losing time, which is a precious variable in some illnesses such as tumours or cancer detection, retinal diseases [59], nodules, etc. 

Since X-rays were invented, imaging, laboratory analysis, and current device technology have been present in the medical field. Still, it must be conceived as a detonating fuse integrated into people’s lives. It must be used on another scale, saving time and resources even if it seems to be a significant investment in new technologies and training people. AI emerges to help people to use technology from a different perspective. It must not continue being only a tool for diagnosing and curing but mainly a source of monitoring and preventing illnesses that is used by physicians. This also guides a paradigm change and conception of technical and technological improvements. It must be seen as an instrument enabling better medical results since decision making settles on daily generated data, helped by devices and the process of making the data [60]. Decision making becomes a process deriving from a way of being. This new method can reduce the mistake rate because of the organisation of those data can improve medical *praxis* and increase patient diagnosis.

Collecting and analysing data will provide the opportunity to understand and prevent patients’ possible reactions to treatments and medications. Health care would be improved and prevention would prevail over healing. But, as we have said before, information accumulates daily, everywhere, which makes their processing difficult. Nowadays, people need tools for a better approach to tackle all emerging situations, for example, new illnesses, physical and mental ones, a lack of food supply, people migration rates, and environmental decline. And, at the same time, this allows AI to drive all the processes. Humanity has developed technology and has worked on it to improve functioning, profits, and to facilitate in day-to-day activities. This reinforces Deleuze’s idea about the social anticipating technical, not inverted [61]. However, despite its origin as a product created, invented, and originated by humans, it is still untrustworthy currently, although it could be worthy of trust.

On the contrary, AI gives place to distrust. This contradicts human behaviour since technology is founded on complex technical and mathematical knowledge that, in principle, will reduce mistakes compared to human activity. In this sense, humanity has seen its benefits. Nevertheless, it is not ready to let it pass before humans, despite the evidence it has offered during these years when it has developed substantial advances that may be used in addition to human interaction, instead of as a tool used by them. But here, as we said before, a cultural change is needed. The idea that humans are superior to all is not necessarily correct. Humans feed machines and AI has been developed by the human hand, and “much more can be achieved compared with the limitation of human labour” [62]. AI facilitates human work and potential human capacities.

At this point, thinking of technology from a different level is necessary. This is the law of progress and technology has been an area of high-speed improvement. Still, we must recognise this replacement, similar to Kurzweil’s Law of Accelerating Returns; he considers there will be times when we go back in progress due to speed and cost-effectiveness, and that should be taken into account [63]. People are not the same either, and in the measure that new generations arrive with other competencies and needs for using technologies, people’s conception must change. Technology, for better or worse, will not slow down the path travelled, and it will continue its tendency to be improved in gigantic steps.

Big data has been, for years, a solution to those data amounts. Nevertheless, artificial intelligence, as mentioned by Ahuya, “has a role to play in medicine as a partner” [64], defining the kind of artefact to use even if it is still a suspicious element in terms of trusting it in many aspects such as confidentiality, being sure that it will do what it is expected with the best results, and misperceptions about its prevalence over humans. 

Nevertheless, the integration must be settled to represent another way of conceiving technology, allowing it to help reduce costs, time, and human mistakes, which will save lives at a higher rate than it is reaching nowadays in developed countries with access to standard technologies and common methodologies for health treatments. Consequently, progress in those countries still finding paths to advance in any area would benefit more quickly from these new applications and visions.

Everybody talks about AI, but what does it mean? Is humanity still far from its current access? What is the reason for creating something without allowing its intervention as far as it was conceived? What is the goal of creating a simulation of the human mind if humans only use it in some of its potentials? Talking AI refers to many computerised processes, from elemental ones to others with a higher complexity. Still, they facilitate human life in all cases, replacing humans in some activities with their “superintelligence” [65]. This is the first obstacle the AI finds, giving place to humans’ fear regarding their employment, change in their quality of life, and others.

However, a machine will never replace people; it will increase their potential and capacities, enabling speedier and less mistaken decision making because of the time provided by the entire system, which allows focus on more critical functions. AI does not work alone; it is required to conform according to people’s needs, who are the ones that select those data to work with, and to be interrelated with others to get the correct information in a short period.

Nevertheless, there are significant differences to consider when referring to AI which indicates the level of integration in a system, whatever it is. This goes from the basic ones such as machine learning to a complete AI system because, as Oracle establishes: “machine-learning is AI, but not all the AI is a machine-learning since the machine learning is focused on creating systems that can learn and improve their performance according to data consumption. The AI focuses more on processes and the superpower think capacity and data analysis than on a specific format or function” [66].

The presence and progress of technology is parallel people’s evolution. As [17] said: “Tools always presuppose a machine, which is always social, before being technical. A social machine always selects or assigns the technical elements used. A tool remains marginal, or little used, until a social machine or collective assemblage exists, which can take it into its ’phylum’”. A health crisis allowed an abrupt positioning in societies, and as [67] says: “technologies are ways of life”. Technological and social advances must be analysed holistically and not separated as it is when discussing technology. Technology in Deleuze’s multiplicities would represent that there is indeed a ‘between’, a set of relations that are not separable [17]. In this sense, the attention is on the connection and interrelation between and among different elements in various processes and stages. 

AI’s potential allows a quicker analysis to treat information compared with humans’ capacity, making the technology more accurate and capable of managing enormous amounts of data. That information has become impossible to achieve by the human mind, and the necessity of technology and new ways of approaching it will enable new procedures in many areas, including Medicine. AI is an ally in this era, and its use in this field represents a better integration of those two spheres: the social and the technological/technical, as explained above. AI would allow the body’s action as a perfect symbiosis between technology and the natural human body’s functions, according to its needs, defining the kind of artefact to use. 

The use of AI in Medicine could improve, more and more, prevention, attention, and healing. It will enable us to anticipate how many patients will suffer a specific illness according to historical clinical data and which ones will develop severe symptoms and even die because of their ailment. It will also provide a more centred treatment to avoid it, even if all patients are unique. This idea follows Aristotle’s’ about difference: “Different things differentiate themselves only through what they have in common” [68]. AI provides the tools to act in the prevention phase. The capacity of AI to work with enormous amounts of data turns it into the best solution for improvement in a delicate area working with human lives. It will exponentiate people’s functions, enabling Deleuze’s “revolutionary becoming”. 

A becoming that is translated to all the areas a human can be related, even more talking about the body since its “becoming” is not static but dynamic all the time, and as Deleuze expressed regarding any concept: “There is nothing that does not lose its identity […] when the dynamic space and time of its actual constitution is discovered” [68], which is another reason to apply AI in this area and involve organisational ways to work with it. It will allow daily communication on patients’ health by “building blocks” [68] (a concept which allows us an image to visualise the categorisation and ordering of those data to make them useful according to what people need and want to reach) oriented to understanding all the elements disturbing the balance in a body. In this sense, the human being works like a phylum of elements, integrated mainly by the body parts and those devices that could be used or even be worn, considering the position that their creators can assume, who use them, such as physicians or technicians and the patient, and how they are constantly changing.

### 4.2. Increasing the Use of Artificial Intelligence in Various Medical Areas

When discussing AI in Medicine, we tend to focus on current areas where it has been used more frequently, as mentioned before, such as imaging for detecting breast cancer, in which “screening augmented with intelligent technology defeated clinical radiologists, showing a lower misdiagnosis rate and a workload decrease of 88%”, for example. However, during the last few years and despite its slow acceptance in some areas, it has also been present in many other fields, such as pathology, eye disease, deontology, dermatology, and ophthalmology [69], with all of them as significant as others areas presumed relevant because of the urgent character of action, as it is cardiology, cancerology, neurology, or metabolic illnesses that jeopardise peoples’ lives when an imbalance takes place. 

In pathology, for example, the speed for analysing and comprehending data still takes too much time between the biochemical test and the results. In this sense, AI intervention in reading considerable data amounts and organising, but mainly analysing them, allows quicker diagnosis and treatments to begin and save time. An example is the possibility of getting histological results in real-time tissue biopsy [70]. As mentioned, “AI is the next step and future of precision pathology and has proposed a new blueprint. The application of AI in pathology has shown bright prospects for predicting a diagnosis. During the analytical work, with augmentation of the AI algorithm, pathological image segmentation, tumour identification, and metastasis determination have been promoted, and the work is finished in higher quality and a shorter period” [70].

#### 4.2.1. Metabolic System

Another example settles in the metabolic field, which includes devices constantly measuring glucose levels used in the metabolic sphere, but patients still prefer to measure it themselves. Using the device will enable the physician to take a better data reading and to know the patient’s actual condition based on many readings, not only the one provided by laboratory results indicating the results from three months ago or an isolated test. This will allow undertaking longitudinal panel or cohort studies, allowing historical results and a better intervention considering previous cases and sequencing analysis. Nevertheless, the patient must cooperate and not panic due to constant sugar level changes. 

The patient must be used to and learn to interact with the device. At the same time, it will benefit the patient better if the device provides insulin doses when necessary as it would avoid patients’ doubt or fear. With such device, physicians and patients could undertake the more accessible part, the following up, and the computer could undertake the more complex work: to provide the needed quantity of drug and the moment of delivering it, with both actions avoiding the imbalance of the patient’s body and health. At the same time, this quicker action will prevent further consequences from imperfect monitoring or severe crises affecting other organs. In this sense, additional research helped by the neuro-fuzzy system has been implemented for a better diabetes diagnosis [71]. One of the benefits of computer aid systems in this field has been the detection of eye diseases such as diabetic retinopathy and glaucoma [72].

#### 4.2.2. Neurological and Mental Areas

In the neurological sphere, another use of AI is to detect neurological symptoms and attack them, such as epilepsy, Alzheimer’s, or dementia conditions. In the first case, the device could avoid seizures and the possibility of death due to drowning if, as a pacemaker does, it acts when the electrical impulse takes place and neutralises the discharge. We must consider that according to the World Health Organization (WHO), there are 50 million people who suffer from these conditions [73].

The device, in this case, will allow real-time reading to provide monitoring of the patient, the number of possible epileptic crises during a specific period, or the number of them, and to intervene, when necessary, while also avoiding patient’s suffering during the emergencies and afterwards, even making it imperceptible for the patient because of the intervention of the device. At the same time, it would provide physicians with enough information to know the illness degree, such as the kind of seizures the patient is suffering from, for a better treatment focused on the specific problem. At present, the Embrace bracelet has been presented to detect epileptic attacks. The bracelet “measures movement and physiological symptoms and alarms those in charge of the person suffering epilepsy” [74].

In dementia and Alzheimer’s conditions, different methods exist to push the brain; for example, a magnetic stimulus is used to make it remember and delay sickness progression and even a magnetic transcranial stimulation can be used to tackle Asperger’s, depression, anxiety, and neurological disorders, severe or not; however, this could derive in long term damage. The Central Nervous System will be protected, as much as possible, if it receives a kind of stimulus, slowing down its degeneration [75]. It also must be considered that all brains are different and that attention must be personalised. Nevertheless, other devices working with AI can be used in earlier stages of Alzheimer’s or dementia diseases as quick detection and knowing the degree of the illness and the phase in which it is settled can provide better follow-up based on long monitoring periods and the possibility of intervention in each degenerative step. This can give a better understanding of the illness and a better patient quality of life. Since the United States approved the first drug to tackle Alzheimer’s, AI could also provide information about when it is necessary to start consuming it and measure doses according to the patient’s needs, even discharging it by monitoring and supplying drugs devices. Machine learning techniques also detect Parkinson’s disease or depression issues [76,77].

#### 4.2.3. Cardiovascular Sphere

Another field in which technology and biosensors are used is the cardiovascular sphere, which is considered the most important for patients’ lives because of affectations on blood vessels and the heart (as it has been forecasted that by 2030 cardiovascular disorders cases will continue to represent the leading death cause.) [78]. In this sense, pacemakers have dominated this area, since the first one was implanted in 1958, intervening while necessary not only to provide an electric reaction but also constant tracking of the sinus-atrial node and frequency modulation; however, the device still needs to be perfected since there are still problems [79]. 

Additionally, there are increasingly more wearable smart devices for the body and clothes, allowing better intervention in diagnosing and monitoring cardiovascular diseases from which physicians and patients may benefit. Even if, at this point, populations must still learn and get used to them until their use becomes a natural clinical practice [80]. And, even if they need improvements in their measures, they can help prevent heart disease since they allow the acquisition of knowledge about heart rate in rest and activity [80]. This will be adequate patient daily habit information that physicians could use to provide clinical attention faster and better and follow the patient to avoid severe health issues in their cardiovascular system since physical activity helps to prevent cardiac outcomes [81]. 

**Figure 2:**
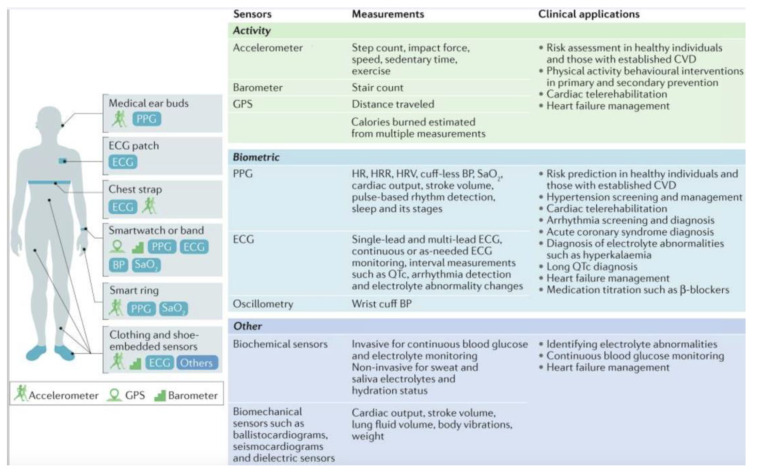
Different smart wearable devices and their cardiovascular applications.

At the same time: “Wearable data also facilitate the application of real-time behavioural change techniques (BCTs) such as just-in-time adaptive interventions, designed to dynamically assess user needs and provide the appropriate amount and type of intervention at the relevant time. Several trials were designed to assess the benefits of wearable-guided BCTs” [80], mainly atrial fibrillation, in which detection and intervention devices with more extended monitoring might be preferable [81] since “now, wearable technology includes physiological and health-care focused measures” [82], as can be seen in Figure 2 [80]:

Other biosensors have been developed recently to prevent, diagnose, and react in different body parts, such as tissues, cells, and the heart. We are in the presence of nano-sensors and nanomedicine [78]. The advantage of these new applications and uses is that they are not only to diagnose or monitor symptoms to prevent severe conditions. Nanomedicine also provides drugs as needed with less invasive procedures and prevents the development of other extreme cardiovascular conditions. For example, nanomedicine can even correct altered circumstances such as revascularisation or cardiac insufficiency, even if it still needs more research [78]. 

## 5. Discussion

The paper contributes to understanding the importance of technology in our daily lives in an area that is the most important to any human being: their health, as opposed to ignoring its use or continuing to use it in a limited way with artefacts and elements provided to facilitate diagnosis but without considering the benefit for everyone, including patients and physicians, of being able to treat the considerable amounts of data generated by each patient. In this sense, focusing on accessing more sophisticated biosensors using artificial intelligence and introducing them as a cultural change in patients and physicians to follow up their health monitoring and diagnosing quality will provide better and more efficient clinical treatment and avoid severe consequences. This will reduce mortality and morbidity rates, mainly in those populations affected by degenerative illnesses or ones with the possibility to lead to death. 

In this sense, we discovered that even if their use has been extended to some medical areas that were not focused on using them, such as ophthalmology, others prevail in their use and have even modified the kind of biosensors. This is the case of imaging and cardiovascular affectations because, on the one hand, they can lead to death abruptly; on the other hand, they were pioneers in using them, such as the pacemaker which monitors and reacts when needed. 

The expanding use of biosensors, from those that provide a reading to those reacting, both for external and internal use, needs a different data and self-care culture from physicians, technicians, data organisers in clinics, and patients. Wearable devices must also be considered valuable tools to manage people’s quality of health and not just for monitoring physical activity as they are currently interpreted and used.

A change in data culture also needs increasing knowledge to work with data for decision making. It demands an innovative culture in clinics and consulting rooms to understand that artificial intelligence provides the capacity to analyse amounts of data that people cannot, and what is the most important in this regard is that data could even be available and shared among many health instances, which will reduce diagnosis and treatment times. Getting data is useless if it cannot be read, interpreted, and used efficiently to provide accurate treatment and even to save lives. This paper considers AI an ally in following up and treating health issues in contemporary societies. Technology is there; humans must use it for the most significant benefits. 

## 6. Conclusions

The human body is a complex system; even if it is possible to separate each into fields and specialities, it is whole. All the systems are interrelated, which affects their function. This is why new technologies need to provide information about everything involved in each activity: organs and what is situated and reached at a distance, cells, body tissues, blood vessels, and other organs. 

As we have seen, biosensors are a familiar implementation since some have over fifty years of experience as pacemakers in cardiology or those used in imaging, to mention two examples. However, they have evolved and increased their presence in other medical branches, such as dermatology or ophthalmology. They also present innovations according to current needs, as is the case of pacemakers or scanning images for better and faster results for diagnosing. Even if there are fields more identified as showing progress, Medicine presents significant advances using technology.

Nevertheless, we consider that what is now needed to benefit more people is to leap to other advanced technologies and techniques that are less invasive for the patient and more effective in helping the physician diagnose and monitor patient symptoms. We propose a more intensive use of machine learning to detect and classify diseases and introduce more and more the use of AI in reading and analysing all the emerging data for faster intervention and to allow prevention more than healing illnesses. Machine learning helps to introduce more and more AI in the sense that it will enable it to work with more specific and advanced technologies which can even be part of the patient to indicate abrupt changes in their daily monitoring. AI will allow us to treat those data amounts. Physicians, in their daily work, cannot process all the information emerging manually; in that sense, advanced technology helps. This is why we propose, as a first step, to work from mapping the way to diagnosing specific symptoms and diseases, to know the organs involved in its correct functioning, cells affected, vessels, etc., considering that they are like “stops” in which the condition can send an alert to the patient and the physician. 

Recognising what is happening or provoking impaired functioning could be more accessible and faster detected. Under this consideration, the next step is to depend on the possible information about consequences in all those “stops” from a biosensor, previously categorised according to their reach and use, which can help to classify symptoms or diseases, detect abnormalities or reactions to a failure in the system studied such as electrical impulses, or even provide drugs. At the same time, it could quantify the number of failures detected to anticipate the severity of the disease or the symptoms to give an idea of when to act.

Wireless capsules and nano-sensors can detect functioning errors and significant changes with holters measuring blood pressure or arrhythmias and electric reactions, allowing better attention by a computer-aided diagnosis that recognises failures and abnormalities. Lots of information already exists and the amount grows constantly. Medicine needs tools to process it faster and more accurately than the human mind can. Human beings need technological and technical inventions to encourage progress for the betterment of humanity, at least for the health of human beings. 

It is time for a change of mind and culture, giving place to data analysis, visibility, and proper and beneficial use. It is time to embrace a “revolutionary becoming” with AI to better the quality of life aided by machines and technology, allowing Deleuze’s evolution and following his social–machine process, considering that the human being precedes the device. In this sense, technology represented by biosensors must constitute that “between” referred to at the beginning of this paper and portray the intermezzo between the patient and the physician. 

It is necessary to think of technology at a different level and to conceive it as an extension of human activity, more than just a human tool for gaining more efficient time management. Technology has been an area of high-speed improvement, but we must recognise this replacement. Technology, for better or worse, will not slow down the path travelled and it will continue its tendency to be improved.

This interrelation between human beings and machines cannot and must not be dissociated but instead deepened and exploded to benefit people’s quality of life.

## Figures and Tables

**Table 1:** EU-supported initiatives concerning activities that involve the use of big data in public health in Europe from 2012 to 2018, in chronological order.

Project Acronym	Full Title	Coordinator Country	Start Date	End Date
FATE	Fall detector for the elderly	Spain	1 March 2012	31 May 2015
MIRRI	Microbial resource research infrastructure	Germany	1 November 2012	30 April 2016
EXPOSOMICS	Enhanced exposure assessment and omic profiling for high priority environmental exposures in Europe	UK	1 November 2012	30 April 2017
ADMOS	Advertising monitoring system development for outdoor media analytics	Hungary	1 September 2013	31 August 2015
DRIVE-AB	Driving re-investment in R&D and responsible antibiotic use	Sweden	1 October 2014	31 December 2017
MARIO	Managing active and healthy aging with use of caring service robots	Ireland	1 February 2015	31 January 2018
METASPACE	Bioinformatics for spatial metabolomics	Germany	1 July 2015	30 April 2018
ComPat	Computing patterns for high performance multiscale computing	Netherlands	1 October 2015	30 September 2018
City4Age	Elderly friendly city services for active and healthy ageing	Italy	1 December 2015	30 November 2018
i-PROGNOSIS	Intelligent Parkinson early detection Guiding Novel Supportive Interventions	Greece	1 February 2016	31 January 2020
ROADMAP	Real world outcomes across the AD spectrum for better care: multimodal data Access Platform	UK	1 November 2016	31 October 2018

## Data Availability

No new data were created or analysed in this study. Data sharing does not apply to this article.
